# The clinical and functional significance of c-Met in breast cancer: a review

**DOI:** 10.1186/s13058-015-0547-6

**Published:** 2015-04-08

**Authors:** Colan M Ho-Yen, J Louise Jones, Stephanie Kermorgant

**Affiliations:** Department of Cellular Pathology, St George’s Healthcare NHS Trust, Blackshaw Road, Tooting, London, SW17 0QT UK; Centre for Tumour Biology, Barts Cancer Institute, Charterhouse Square, London, EC1M 6BQ UK

## Abstract

c-Met is a receptor tyrosine kinase that upon binding of its ligand, hepatocyte growth factor (HGF), activates downstream pathways with diverse cellular functions that are important in organ development and cancer progression. Anomalous c-Met signalling has been described in a variety of cancer types, and the receptor is regarded as a novel therapeutic target. In breast cancer there is a need to develop new treatments, particularly for the aggressive subtypes such as triple-negative and basal-like cancer, which currently lack targeted therapy. Over the last two decades, much has been learnt about the functional role of c-Met signalling in different models of breast development and cancer. This work has been complemented by clinical studies, establishing the prognostic significance of c-Met in tissue samples of breast cancer. While the clinical trials of anti-c-Met therapy in advanced breast cancer progress, there is a need to review the existing evidence so that the potential of these treatments can be better appreciated. The aim of this article is to examine the role of HGF/c-Met signalling in *in vitro* and *in vivo* models of breast cancer, to describe the mechanisms of aberrant c-Met signalling in human tissues, and to give a brief overview of the anti-c-Met therapies currently being evaluated in breast cancer patients. We will show that the HGF/c-Met pathway is associated with breast cancer progression and suggest that there is a firm basis for continued development of anti-c-Met treatment, particularly for patients with basal-like and triple-negative breast cancer.

## Introduction

The receptor tyrosine kinase (RTK) c-Met was originally identified as the product of a transforming gene generated from a chemically transformed osteosarcoma cell line [1]. In 1991, c-Met was discovered to be the receptor for hepatocyte growth factor (HGF), a protein that had previously been shown to promote hepatocyte growth in culture [2,3]. Mutations in the *MET* gene were subsequently described in hereditary and sporadic papillary renal cell carcinomas [4]. Since then, dysregulation of c-Met signalling has been identified in a variety of malignant and premalignant lesions, including those arising in the breast, lung, stomach, pharynx, colorectum and cervix [5-10]. Accordingly, the utility of targeting c-Met in different cancer types is now being evaluated in clinical trials [11].

New therapeutic targets are needed in breast cancer, particularly in patients with triple-negative (TN) breast cancer and the related basal-like (BL) subgroup of breast cancer. Although distinct, BL tumours can be considered an aggressive subgroup of TN cancers, and both are characterised by a lack of oestrogen receptor and c-erbB2 (Her2) expression, limiting systemic treatment options [12,13]. Since their discovery, the literature regarding c-Met and HGF in the breast has grown rapidly, and there is now a need to consolidate the findings from these studies to better understand the relevance of anti-c-Met therapy in breast cancer.

The aim of this review is to explore the roles of HGF/c-Met signalling in breast development, different *in vitro* and *in vivo* models of breast cancer, and the various mechanisms of aberrant c-Met signalling identified in breast cancer tissue. We will also outline the anti-c-Met compounds currently being investigated as possible breast cancer treatments.

## Structure and function

c-Met is first produced as a 170 kDa precursor that then undergoes proteolytic cleavage, generating a 50 kDa α-subunit and a 145 kDa β-subunit [3,14]. The extracellular α-subunit is attached to the transmembrane β-subunit by a disulphide bond (reviewed in [15]). A Sema domain, a PSI domain (so-called because it is present in plexins, semaphorins and integrins) and four IPT domains (immunoglobulin-like fold shared by plexins and transcription factors) make up the extracellular portion of c-Met. The intracellular aspect contains three further domains: the juxtamembrane region, which is important in downgrading kinase activity following Ser 975 phosphorylation; the catalytic domain that harbours the Y1234 and Y1235 residues; and the multifunctional carboxy-terminal docking site [15].

The only known mammalian agonistic ligand for c-Met is HGF (also known as scatter factor) [16]. As is the case with c-Met, HGF is secreted first as a precursor, which must then be activated by proteases, resulting in the formation of a mature heterodimer composed of an α-chain and a β-chain [17].

When HGF binds to c-Met, the receptor undergoes autophosphorylation of the Y1234 and Y1235 residues in the kinase domain [14]. Subsequently, tyrosine residues in the docking site (Y1349 and Y1356) become phosphorylated, permitting binding of adaptor molecules including growth factor receptor-bound protein 2, growth factor receptor-bound protein 2-associated binder 1 and Shc [14,15]. These molecules facilitate downstream signalling through several pathways, such as the Rac1/Cdc42 pathway, the phosphoinositide 3-kinase/Akt pathway, signal transducer and activator of transcription 3 and the Erk/mitogen-activated protein kinase cascade [15,18]. Together, these pathways regulate cellular proliferation, motility, migration, invasion and tubulogenesis [18].

The only other ligand known to bind c-Met in mammals is decorin, a leucine-rich proteoglycan [16]. Decorin has been shown to antagonise c-Met signalling by promoting intracellular degradation of the receptor, resulting in suppression of c-Met-mediated cell migration and growth [16].

**Figure 1:**
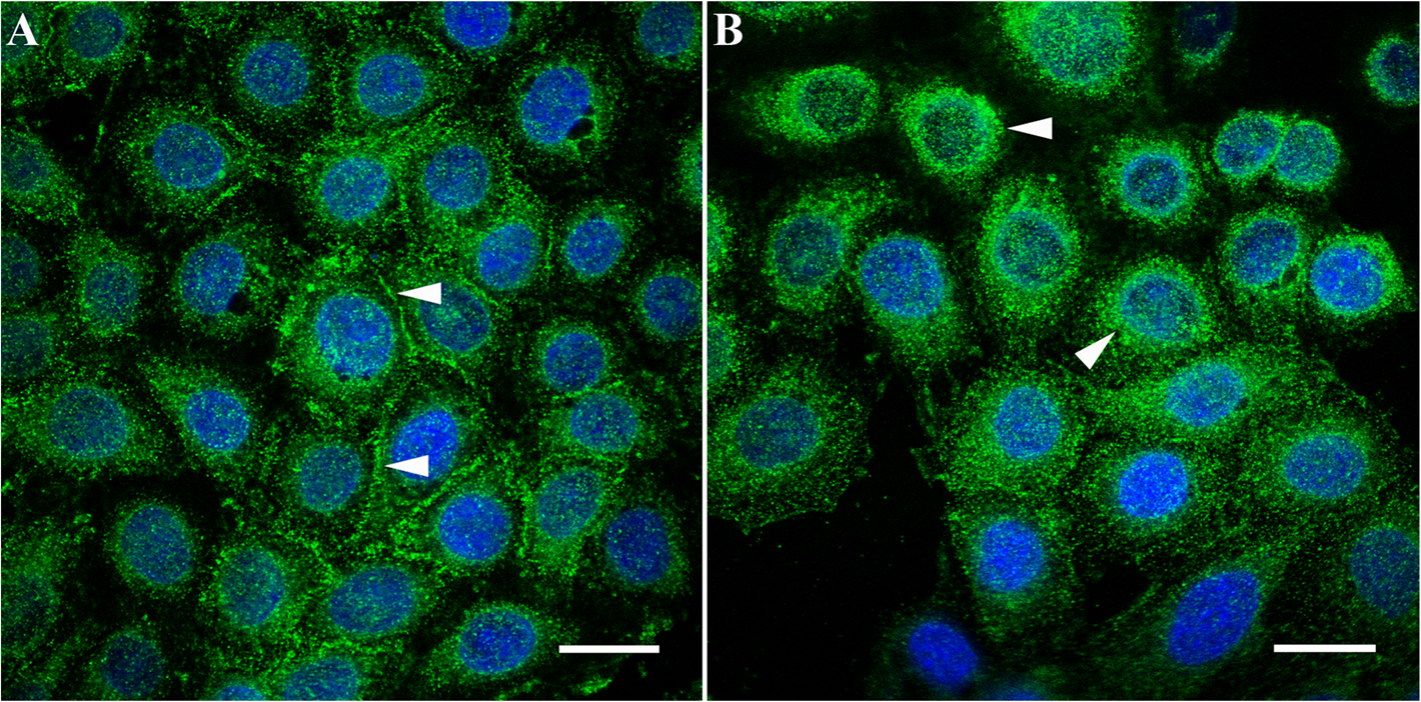
**Trafficking of c-Met in MDA-MB-468 cells. (A)** In resting cells there is prominent membrane expression of the receptor (white arrowheads). **(B)** Following hepatocyte growth factor stimulation there is internalisation of the receptor and a predominantly perinuclear, granular pattern of staining (white arrowheads), consistent with the presence of c-Met within endosomes. Immunofluorescence (green, c-Met; blue, nuclei); ×63 objective under oil immersion. Scale bars represent 20 μm.

In common with other RTKs, c-Met is regulated by the ubiquitin ligase, Cbl [19,20]. Following c-Met activation, phosphorylation of the Y1003 residue in the juxtamembrane region recruits Cbl to c-Met, permitting polyubiquitination and degradation of the receptor [19,20]. Although c-Met internalisation is part of the process of signal attenuation, trafficking of the receptor within endosomes, under the control of protein kinase C, results in sustained signalling and is necessary for HGF-mediated migration [21-23] (Figure 1).

## Hepatocyte growth factor/c-Met signalling in breast development

The relationship between HGF and c-Met during development is complex and dynamic. In a study of mouse embryos, Andermarcher and colleagues described a shift in expression of HGF and c-Met from gastrulation to early organogenesis [24]. It was noted that while HGF and c-Met were coexpressed in endodermal and mesodermal cells during gastrulation, the onset of organogenesis coincided with HGF being localised to the mesenchyme and c-Met to the epithelium and endothelium [24]. The authors suggested that this change from an autocrine to a paracrine relationship may reflect the different roles of the pathway at different stages of development [24].

The HGF/c-Met pathway may also have distinct roles in the different compartments of the mammary epithelium. Exposure of luminal epithelial and myoepithelial cells to HGF evokes contrasting effects in the two cell types [25]. The growth rate of luminal cells increased fivefold to ninefold with HGF compared with controls, and no morphological changes were seen. In contrast, HGF had no effect on myoepithelial cell growth but did induce extensive branch formation [25]. RT-PCR analysis showed c-Met expression was higher in luminal cells than in myoepithelial cells, which may explain these differences. The authors of this study considered the developmental relevance of this arrangement, and hypothesised that the myoepithelial cells lay down the ductal framework that the luminal cells proliferate and migrate along, thus populating and extending the ductal system [25].

Other studies utilising primary murine mammary epithelial cells in *in vivo* models have further emphasised the importance of the HGF/c-Met pathway in mammary development [26,27]. Overexpression of HGF in primary murine mammary epithelial cells and subsequent transplantation of these cells into the cleared mammary fat pads of mice resulted in a marked increase in ductal branches/bifurcations, along with an increase in the size and number of ductal end buds [26,27]. Importantly, immunohistochemistry revealed an increase in basal/myoepithelial marker expression (smooth muscle actin, cytokeratin 14 and p63) and a reduction in luminal marker expression (cytokeratin 18 and oestrogen receptor) compared with control mice [27]. This finding led the authors to suggest that c-Met signalling directs progenitor cells towards a basal phenotype over luminal differentiation [27], which is reflected in the pattern of c-Met staining seen in different breast cancer subtypes (as discussed later).

## Aberrant c-Met signalling in breast cancer

A broad range of mechanisms may result in aberrant c-Met signalling, including activating gene mutations, gene amplification, protein overexpression, increased ligand-dependent paracrine stimulation and the acquisition of autocrine signalling [28] (Table 1). In breast cancer, the majority of studies have looked at the significance of protein overexpression of c-Met and the relationship between levels of the receptor and prognostic factors/survival.

**Table 1:** **Mechanisms of aberrant c-Met signalling in invasive breast cancer**

**Mechanism**	**Frequency/prognostic significance in breast cancer**	**Reference**
Gene mutation	*MET* mutations are uncommon; *HGF* promoter region mutations occur in 15 to 51% of breast cancers	[30,31]
Gene amplification	*MET* amplification is uncommon, occurring in 0 to 8% of breast cancers; *MET* copy number is positively correlated with TN tumours	[32,33]
	Patients with trastuzumab-treated Her2-positive metastatic breast cancer show *MET* amplification in 27.7% of cases and *HGF* amplification in 39.3% of cases; patients with *MET*-amplified Her2-positive tumours have a shorter time to progression	[34]
Autocrine signalling	*HGF* and *MET* mRNA detected in tumour cells in all breast cancers analysed, with strongest positivity at the advancing edge of the tumour	[35]
	On IHC, autocrine pattern of staining seen in 46.6% of tumours	[37]
Paracrine signalling	On IHC, paracrine pattern seen in 59.1% of tumours; paracrine signalling is associated with a worse outcome when c-Met staining is more intense at the tumour front	[68]
C-Met activity (phosphorylation)	Using RPPA, 47.9% of tumours showed high phospho-c-Met expression; inconsistent relationship with molecular subtype; high phospho-c-Met associated with an increased risk of tumour recurrence	[43,44]

Frequency and prognostic significance of the different mechanisms of aberrant c-Met signalling in invasive breast cancer, identified in studies using human tissue samples. HGF, hepatocyte growth factor, IHC, immunohistochemistry, RPPA, reverse-phase protein arrays; TN, triple negative.

**Table 2:** **Relationship between c-Met expression and prognostic factors**

**Prognostic parameter**	**Relationship**	**Reference**
Age at presentation	No established relationship	[6,46,49,50]
Tumour size	Most studies have found no relationship	[36,46,49,53]
	We found inverse correlation between c-Met expression and tumour size	[50]
Lymph node status	Most studies show no relationship	[6,49,53]
	We found higher c-Met expression in node-negative tumours	[50]
Tumour grade	Mixed; some studies show no association	[6,36,48]
	Some studies show increased c-Met expression in high-grade tumours	[46,49]
	One study showed increased c-Met in low-grade tumours	[53]
Histological subtype	Increased c-Met in tubular carcinoma, decreased in lobular carcinoma	[50]
Molecular subtype	Increased c-Met in basal-like breast cancer	[50,51,55,82]
Survival	Increased c-Met associated with reduced survival	[6,38,45-51]

**Table 3:** **Anti-c-Met therapies currently under investigation in clinical trials for breast cancer** [11]

**Compound**	**Target/mechanism of action**	**ClinicalTrials.gov identifier**
Tivantinib (ARQ197)	c-Met/non-ATP kinase inhibitor	NCT 01575522
Cabozantinib (XL184)	c-Met and VEGFR, along with RET, KIT, AXL/kinase inhibitor	NCT 01738438
Foretinib (XL880)	c-Met and VEGFR, along with KIT, Flt-3, PDGFR, Tie-2/kinase inhibitor	NCT 01147484
MetMab (onartuzumab)	c-Met/anti-c-Met antibody	NCT 01186991

PDGFR, platelet-derived growth factor receptor; VEGFR, vascular endothelial growth factor receptor.

### Gene mutation

Following the discovery of mutations in the tyrosine kinase domain of *MET* in hereditary and sporadic papillary renal cell carcinomas [4], *MET* mutations have also been found in up to 30% of cancers of unknown primary origin [29]. These mutations include those in the SEMA domain and the juxtamembrane domain and an activating mutation in the tyrosine kinase domain [29]. However, few studies have assessed the frequency of *MET* mutations in primary breast cancer. In a small study comprised of 11 patients with breast cancer (including six patients that showed loss of heterozygosity in the region of the *MET* gene), no mutations in the tyrosine kinase domain of the *MET* were identified [30], suggesting that this is not a common event in breast cancer. In contrast, a mutation in the HGF promoter region referred to as the deoxyadenosine tract element appears to be a frequent event, having been identified in 15% of European breast cancer patients and over 50% of African Americans with breast cancer [31]. A truncation mutation in the deoxyadenosine tract element activates the HGF promoter in breast cancer cells, leading to the formation of an HGF/c-Met autocrine loop [31].

### Gene amplification

Amplification of the *MET* gene (located on chromosome 7), like mutation, is unusual in invasive breast cancer: in a study of 155 patients, Carracedo and colleagues did not identify *MET* amplification at all (although 22% of tumours showed low-grade polysomy) [32]. Elsewhere, in a much larger study, Gonzalez-Angulo and colleagues found increased copy numbers of *MET* in a minority of cases (82 out of 971 tumours studied) [33]. Although a high copy number of *MET* was not an independent predictor of recurrence-free survival, these workers did note lower recurrence-free survival rates in the *MET-*amplified group on univariate analysis [33]. Moreover, there was a positive correlation between *MET* copy number and TN status [33].

Amplification of *MET* may also be important in other molecular subtypes of breast cancer: in a study of 130 Her2-positive breast cancers, both *MET* and *HGF* amplification were associated with trastuzumab failure and patients with *MET* amplified tumours had a shorter time to progression [34].

### Autocrine/paracrine signalling and c-Met activation

Several lines of evidence suggest that HGF-dependent c-Met signalling (both paracrine and autocrine) is an important mediator of breast cancer progression [35-38]. HGF and c-Met are frequently coexpressed in invasive breast cancers: c-Met in epithelial cells and HGF in epithelial cells (autocrine pattern) and/or stromal cells (paracrine pattern) [35-37]. HGF/c-Met coexpression is often strongest at the infiltrative margins of tumours [35,37]. Moreover, when tumours demonstrate this strong coexpression at the advancing edge, there is a significant correlation with high tumour grade, an increased proliferation index and reduced survival, compared with cancers that are negative for coexpression [37]. Expression of matriptase (an activator of HGF) is positively correlated with both c-Met and HGF in invasive breast cancer, and high levels of c-Met and matriptase are associated with reduced 30-year survival at univariate analysis [38].

While analysis of HGF/c-Met coexpression provides useful insight into the role of ligand-dependent c-Met activation, measuring c-Met phosphorylation would also take into account ligand-independent c-Met activation, theoretically giving a more global readout of c-Met signalling. Unfortunately, the detection of c-Met phosphorylation in human formalin-fixed, paraffin-embedded samples is complicated by the poor stability of phospho-epitopes in general [39,40] and by the limited sensitivity and specificity of phospho-specific antibodies [41,42]. It is therefore perhaps not surprising that few studies have investigated the prognostic significance of c-Met phosphorylation in invasive breast cancer. Two studies that have managed to identify phospho-c-Met in breast cancer did so using reverse-phase protein analysis, with contrasting results [43,44]. In a study of 107 primary breast cancer patients, Hochgräfe and colleagues found higher phospho-c-Met expression (pY1234/5) in TN tumours [43], whereas Raghav and colleagues found no difference in phospho-c-Met expression (pY1235) between different molecular subtypes but did find higher recurrence rates in patients whose tumours showed high levels of pY1235 [44].

### Protein overexpression

c-Met protein overexpression, as assessed by immunohistochemistry (IHC)/immunofluorescence, is now generally accepted to be a poor prognostic factor in invasive breast cancer [6,38,45-51] (Table 2). Exactly what constitutes overexpression is less clear, and several different scoring methods and cutoff points were utilised in these studies, resulting in a variable proportion of cases being classified as c-Met-positive (15 to 63%) [6,38,45-49,51]. Of course, the characteristics of the study population and the choice of antibody for the IHC assay are additional variables that may influence the proportion of c-Met-positive cases. Two particular issues related to c-Met IHC that deserve further comment are the reproducibility of staining from commercially available antibodies and the domain of the receptor targeted by the antibody.

In an analysis of six different commercial c-Met antibodies (five of which recognised the protein at western blot), Pozner-Moulis and colleagues found a low correlation between c-Met expression on different sections of the same tissue microarrays stained with the same antibody [52]. Moreover, when different lots of the same monoclonal antibody were applied to the same tumour, marked differences were seen in the staining pattern. These findings suggest that many c-Met antibodies may not be providing a reproducible evaluation of c-Met expression [52]. Several studies have also commented on the importance of selecting c-Met antibodies that target the intracellular domain, since expression of this part of the receptor appears to have more prognostic relevance than those directed against the extracellular region [38,47,52]. Thus, there is now a need to develop standardised guidelines for the methodology (such as the use of validated anti-c-Met antibodies) and interpretation of c-Met IHC.

Most studies have found no association between c-Met expression and established prognostic factors, such as age at presentation, tumour size and lymph node status [6,36,46,49,53], perhaps explaining why c-Met expression retains prognostic power after correcting for these factors on multivariate analysis [38,45-48,50]. Interestingly, a large recent study utilising breast cancers from 924 patients did find a positive correlation between c-Met expression and both increasing tumour size and nodal involvement; c-Met-overexpressing tumours were associated with worse survival, but not on multivariate analysis [51]. With regard to tumour grade there is no consensus, with some studies finding no association [6,36,48], other studies finding increased c-Met expression in high-grade tumours [46,49] and one study identifying more frequent immunoreactivity in grade 1 tumours compared with grade 3 cancers (75% versus 43.8%, respectively) [53].

**Figure 2:**
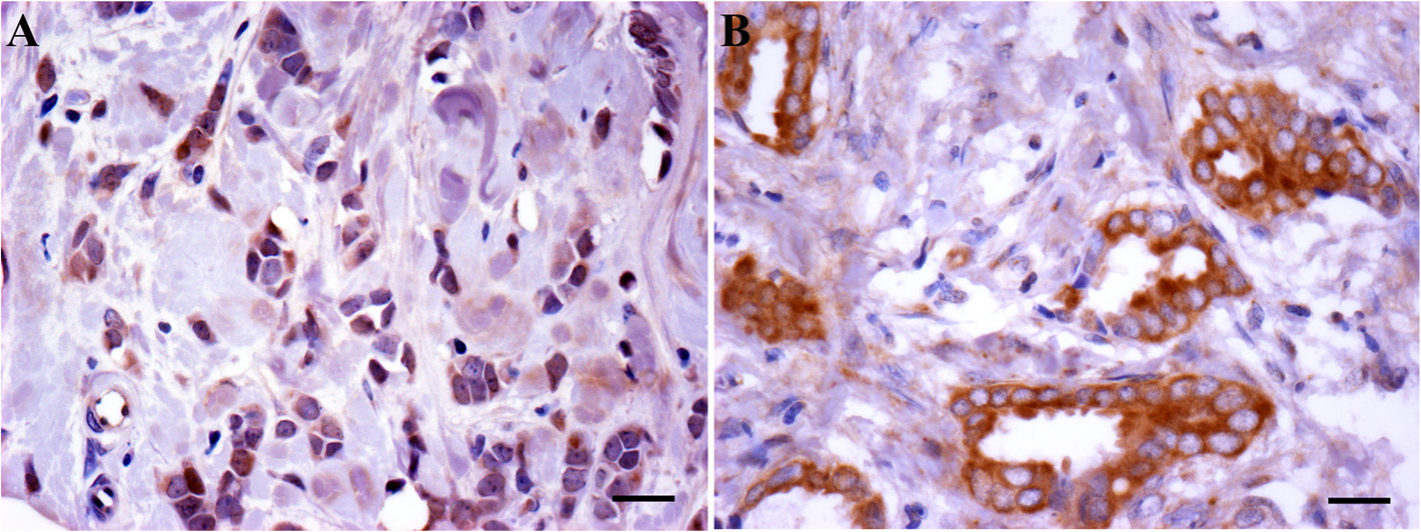
**c-Met expression varies between histological subtypes of breast cancer.** **(A)** Invasive lobular carcinoma characterised by discohesive tumour cells with low c-Met expression. **(B)** Tubular carcinoma with cohesive tumour cells arranged in angulated tubules with strong expression of c-Met. Immunohistochemistry, ×40 objective. Scale bars represent 20 μm.

**Figure 3:**
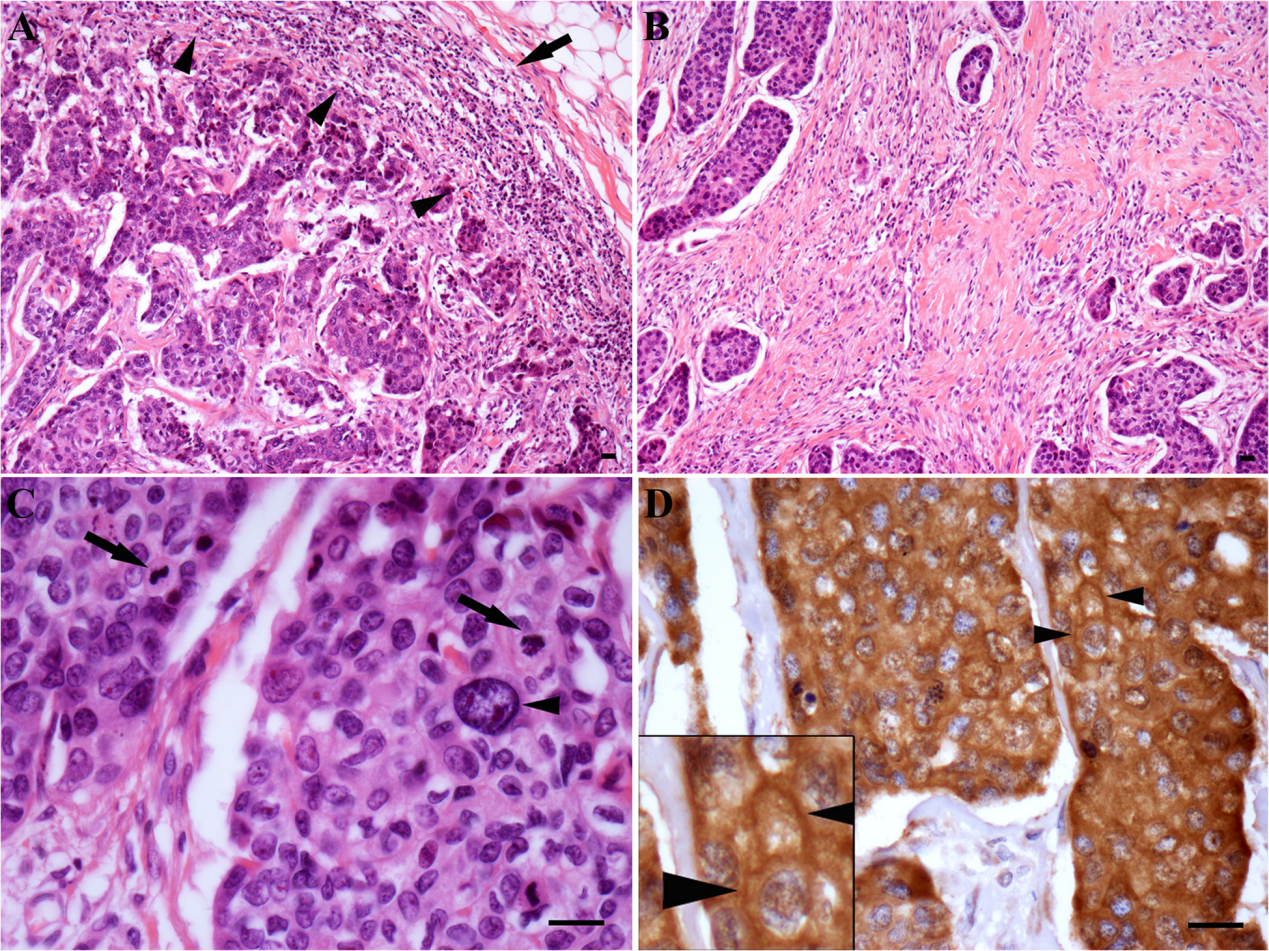
**c-Met expression in basal-like breast cancer.** Characteristic features of basal-like breast cancer (images are from the same tumour). **(A)** Circumscribed tumour front (arrowheads) and associated chronic inflammatory cell infiltrate (arrow). **(B)** Tumour fibrosis. **(C)** High-grade cytology, with nuclear enlargement and pleomorphism (arrowheads), along with prominent mitotic figures (arrows). Haematoxylin and eosin; **(A)** and **(B)** × 10 objective, **(C)** × 40 objective. Scale bars represent 20 μm. **(D)** High cytoplasmic and membranous (arrowheads) expression of c-Met. Immunohistochemistry, ×40 objective. Scale bar represents 20 μm. Inset image is at 200% magnification.

In our own analysis we identified significantly different levels of c-Met expression in two special histological subtypes of breast cancer: levels were lower in the E-Cadherin-negative invasive lobular carcinomas and higher in tubular carcinomas [50] (Figure 2), a well-differentiated tumour subtype characterised by angulated tubules [54]. These observations are reminiscent of findings from the aforementioned studies on mammary development, where HGF stimulated tubule formation in murine mammary epithelial cells [26,27]. We also demonstrated, for the first time, that c-Met protein expression was independently associated with BL breast cancer (Figure 3), a finding supported by the results from the most recent IHC analyses [50,51]. Together these findings indicate that patients with BL cancer should be included in clinical trials of anti-c-Met therapy.

## Hepatocyte growth factor/c-Met signalling in breast cancer cells

A variety of breast cancer cell lines (BCLs) have been used to study the role of HGF and c-Met in a range of cellular processes implicated in the progression of breast cancer. Although these BCLs include those representative of the luminal, Her2-overexpressing and BL subtypes, *MET* overexpression at the RNA level and c-Met protein overexpression are more often seen in the BL BCLs [55,56].

### Tubulogenesis

The extent of tubule formation is a key component of the grading system in invasive breast cancer [57]. A lack of tubular differentiation is a feature of high-grade tumours, which are associated with a poorer outlook than their low-grade counterparts [57]. Tubule formation has been observed in T47D and MCF7 cells in response to HGF treatment and c-Met expression has been identified at confocal microscopy in T47D cells that bordered luminal structures [58]. However, the relationship seems complex. When similar experiments were performed on colon carcinoma cell lines, low doses of HGF (1 to 10 ng/ml) stimulated tubule formation, but higher doses (up to 100 ng/ml) appeared to abrogate this phenomenon [58].

### Migration and invasion

The promigratory and proinvasive effects of HGF have been shown in several BCLs, including MCF7, MCF10.DCIS and MDA-MB-231 [59-62]. Administration of HGF, either in the form of recombinant HGF, conditioned media from HGF-secreting fibroblasts or by way of co-culture with HGF-secreting fibroblasts, has been shown to significantly increase migration and invasion in wound closure and transwell invasion assays [59-62]. In addition, adipose-derived mesenchymal cells isolated from lipoaspirates express variable levels of HGF and co-culture of these cells with MDA-MB-231 cells resulted in increased migration [63]. These studies are supported by work demonstrating that NK4, a variant form of HGF that competitively inhibits HGF binding [64], reduces HGF-mediated c-Met phosphorylation and inhibits HGF-induced scattering and invasion of MCF7 and MDA-MB-231 cells [59].

Numerous studies have sought to uncover the mechanisms through which HGF/c-Met signalling contributes to the migratory and invasive phenotype in breast cancer, particularly focusing on pathways associated with epithelial adhesion [65-67]. E-Cadherin is a key component of adherens junctions (specialised intraepithelial junctions) [68,69], and is regarded by some as a tumour suppressor important in the prevention of cell migration, invasion and metastasis [69]. In MCF7 cells, E-Cadherin and c-Met are co-localised at the cell membrane in regions of cell–cell contact [65,70]. Following treatment with HGF, lysates from these cells show a reduction in E-Cadherin expression, and immunofluorescent studies demonstrate asymmetric accumulation of c-Met and E-Cadherin in the cytosol, ultimately leading to complete internalisation of both proteins after 2 hours [61,70].

In addition to favouring epithelial dissociation via E-Cadherin downregulation/internalisation, there is some evidence that HGF/c-Met signalling contributes to breast cancer progression by promoting cancer cell adhesion to components of the extracellular matrix [66]. HGF treatment increased adhesion of MtLn3 rat mammary adenocarcinoma cells to laminin, type 1 collagen and fibronectin, compared with control cells [66]. Furthermore, treatment with HGF was associated with lamellipodia formation, focal adhesion kinase phosphorylation and focal adhesion kinase expression at focal contacts, suggesting that c-Met and focal adhesion kinase cooperate to promote cancer cell/substrate adhesion [66].

Proteolytic pathway regulation is another mechanism influenced by HGF/c-Met signalling in the *in vitro* setting [60]. Conditioned media from HGF-secreting fibroblasts and recombinant HGF treatment resulted in increased secretion of both urokinase-type plasminogen activator and its receptor (urokinase-type plasminogen activator receptor) by different ductal carcinoma *in situ* cell lines (MCF10.DCIS cells and SUM102 cells). Increased collagen IV degradation was also demonstrated, along with increased numbers of invasive outgrowths in three-dimensional cultures of ductal carcinoma *in situ* cells when in the presence of HGF [60]. Together, these findings implicate HGF-secreting fibroblasts in the progression of ductal carcinoma *in situ* to invasive cancer [60].

### Cell survival

HGF/c-Met signalling has been associated with both pro-apoptotic and anti-apoptotic effects [71]. Using the murine hepatocellular carcinoma cell line Hepa1-6, Wang and colleagues established that c-Met and the death receptor FAS formed a complex; they proposed a model in which c-Met sequesters FAS, thus preventing ligand-independent activation (due to clustering) and FAS ligand/FAS binding, resulting in cell survival [71]. In this model, high levels of HGF (or FAS ligand) would cause dissociation of the c-Met/FAS complex, leaving the cells vulnerable to FAS-mediated apoptosis. Such a model would explain the paradoxical effects of HGF/c-Met on cell survival [71].

A similar mechanism may also exist in breast cancer cells, where treatment of preneoplastic MCF-10AT breast epithelial cells with anti-FAS (an activator of FAS signalling) induced c-Met/FAS complex dissociation and apoptosis [72]. It has also been shown that HGF protects MDA-MB-453 breast cancer cells from adriamycin-induced apoptosis [73]. Preincubation of these cells with HGF blocked adriamycin-mediated FAS ligand upregulation and inhibited the reduction in levels of the anti-apoptotic protein Bcl-X_L_ [73,74].

### Cross-talk with other receptor tyrosine kinases

It is widely appreciated that c-Met can cross-talk with a variety of other cell surface receptors (reviewed in [75]). In breast cancer, cross-talk between c-Met and members of the c-erbB family in particular has received considerable interest. HGF has been shown to trans-activate epidermal growth factor receptor (EGFR) in PyVmT mouse mammary carcinoma cells [76]. Moreover, the EGFR inhibitor gefitinib blocked HGF-mediated proliferation in PyVmT cells, migration in PyVmT cells and NMuMG cells, and invasion in PyVmT cells, NMuMG cells and MDA-MB-231 cells [76]. The authors went on to show that gefitinib effects c-Met activation in an EGFR-dependent process (as opposed to directly targeting c-Met) by finding no effect on c-Met activation when EGFR-null/c-Met expressing haematopoietic 32D cells were treated with the inhibitor [76].

Similarly, another member of the c-erbB family – Her2 – has been noted to cross-talk with c-Met in HCC1954 breast cancer cells, which overexpress c-Met and Her2 [77]. In HCC1954 cells, knockdown of *MET* resulted in increased Her2 phosphorylation and, conversely, knockdown of *Her2* was associated with an increase in c-Met activity [77].

The relationship between c-Met and other RTKs has important implications for the development of resistance to anti-RTK therapies already in clinical use – now a significant problem in breast cancer treatment [77]. Indeed, in the EGFR tyrosine kinase inhibitor-resistant cell line SUM229, c-Met is phosphorylated and thought to stimulate EGFR phosphorylation in the presence of EGFR inhibitors in a Src-mediated process [78]. Likewise, treatment of the Her2-overexpressing BT-474 and SKBR3 cells with trastuzumab upregulated c-Met protein expression in just 48 hours [79]. HGF-mediated c-Met phosphorylation in these cells opposed trastuzumab-mediated growth inhibition by abrogating p27 induction [79]. c-Met therefore plays an important role in breast cancer cell function and signalling by virtue of its ability to interact with other RTKs.

## *In vivo* models of HGF/c-Met-mediated tumour formation

The *in vivo* effects of aberrant HGF/c-Met signalling have been explored in different mouse models [80-83]. Mice harbouring the whey acidic protein *WAP-HGF* transgenic construct show elevated HGF expression in mammary epithelium, compared with wild-type mice, and go on to develop mammary tumours characterised by a high Ki67 proliferation index, a reduced progesterone receptor immunoreactivity and areas of squamous differentiation (a feature of BL breast cancers) [80,84].

Squamous metaplasia was also detected in a high proportion (65%) of mammary tumours that developed in mice with mutationally activated *MET* [82]. The majority of these tumours also expressed the basal cytokeratin, cytokeratin 5 [82]. Elsewhere, Ponzo and colleagues studied transgenic mice that express oncogenic *MET* in the mammary epithelium, under the control of the murine mammary tumour virus promoter [81]. About one-half of the tumours that developed in these mice showed variable histological patterns which included BL features [85] such as squamous/spindle cell differentiation, high nuclear grade, necrosis and lymphocytic infiltration [81]. These tumours also expressed cytokeratin 5/6 and cytokeratin 14 (another basal cytokeratin) on IHC [81]. Interestingly, in a subsequent study these workers found that loss of *TP53*, in addition to oncogenic *MET* expression, was associated with the formation of tumours with a claudin-low profile, a recently described subgroup of TN tumours that is distinct from the BL subtype [83,86].

## Anti-c-Met therapy in invasive breast cancer

There are various strategies for antagonising HGF/c-Met signalling: antibodies can be directed against c-Met; HGF itself can be targeted with antibodies; and the catalytic function of c-Met can be opposed with tyrosine kinase inhibitors, which account for the majority of anti-c-Met compounds under investigation [87]. Four therapies currently in phase II clinical trials for the treatment of advanced TN breast cancer are tivantinib (also known as ARQ197) [ClinicalTrials.gov:NCT 01575522], cabozantinib (alternatively known as XL184) [ClinicalTrials.gov:NCT 01738438], MetMab (onartuzumab) [ClinicalTrials.gov:NCT 01186991] and foretinib (XL880) [ClinicalTrials.gov:NCT 01147484] [11] (Table 3).

As well as breast cancer, the anti-c-Met monoclonal antibody MetMab is also being trialled in lung cancer and colon cancer [88]. Unlike many other anti-c-Met antibodies, MetMab is monovalent – therefore it does not promote dimerisation when it binds to c-Met, thus avoiding the agonistic effects associated with similar therapies [88].

Tivantinib belongs to the c-Met tyrosine kinase inhibitor class of c-Met antagonists, and is a non-ATP competitive inhibitor of the receptor [89,90]. A phase I trial of tivantinib in 51 patients with solid tumours (including two patients with breast cancer) found the inhibitor to be well tolerated, with fatigue, nausea and vomiting being the most common adverse effects [90]. Furthermore, when pretreatment and on-treatment tumour biopsies were compared, there was a reduction in total and phosphorylated c-Met expression in the on-treatment samples, suggesting that tivantinib inhibited intra-tumoural c-Met signalling [90].

Cabozantinib is another small molecule inhibitor of c-Met, which targets a range of tyrosine kinases, including RET, KIT and AXL, but particularly c-Met and VEGFR2 [91]. Cabozantinib not only inhibits c-Met phosphorylation *in vivo*, but also promotes tumour hypoxia and cell death and inhibits the growth of MDA-MB-231 tumours in a dose-dependent manner [91]. It has been suggested that the ability of cabozantinib to inhibit both c-Met and vascular endothelial growth factor receptor 2 may actually counter the c-Met-dependent resistance noted when only the vascular endothelial growth factor pathway is targeted [91].

Foretinib is a small molecule kinase inhibitor that principally targets c-Met and vascular endothelial growth factor receptor [92]. Foretinib inhibits HGF-induced c-Met phosphorylation, inhibits tumour cell growth in hypoxic and normoxic conditions, and has been shown to reduce tumour cell burden in an *in vivo* model of lung cancer [92]. A phase I study in patients with a wide variety of solid organ cancers (including one breast cancer patient) found the inhibitor to be safe and noted a partial response in 7.5% of patients and stable disease in a further 55% of patients [93].

## Conclusion

Much progress has been made in our understanding of c-Met/HGF signalling in recent years, and there is now convincing *in vitro* and *in vivo* evidence that this is an important pathway in mammary development and cancer progression. Clinical studies have confirmed the prognostic significance of c-Met expression in breast cancer and highlight the potential of c-Met inhibitors as a novel form of targeted therapy. The possible role of c-Met signalling in promoting BL breast cancer is noteworthy, and merits further investigation in the experimental and clinical trial settings. The outcomes of ongoing and future clinical trials of anti-c-Met therapy will be eagerly anticipated, but issues such as receptor cross-talk and resistance may need to be addressed if treatment efficacy is to be maximised. It is also important to stratify patients appropriately, and the development of standardised prognostic/predictive assays will be crucial in identifying those subgroups of patients most likely to benefit from anti-c-Met therapy.

## Abbreviations

BCL: Breast cancer cell line
BL: Basal-like
EGFR: Epidermal growth factor receptor
HGF: Hepatocyte growth factor
IHC: Immunohistochemistry
RTK: Receptor tyrosine kinase
TN: Triple negative
